# TMT-labeling Proteomics of Papillary Thyroid Carcinoma Reveal Invasive Biomarkers

**DOI:** 10.7150/jca.47290

**Published:** 2020-08-25

**Authors:** Jiaqi Dai, Xiaqing Yu, Yali Han, Li Chai, Yina Liao, Peng Zhong, Ruting Xie, Xuechen Sun, Qingqing Huang, Jian Wang, Zhiqiang Yin, Yun Zhang, Zhongwei Lv, Chengyou Jia

**Affiliations:** 1 Shanghai Research Center for Thyroid Diseases, Shanghai Tenth People's Hospital, Tongji University School of Medicine, Shanghai, 200072, P. R. China.; 2 Department of Nuclear Medicine, Shanghai Tenth People's Hospital, Tongji University School of Medicine, Shanghai, P. R. China.; 3 Department of Pathology, Shanghai Tenth People's Hospital, Tongji University School of Medicine, Shanghai, P. R. China.; 4 Department of Nuclear Medicine, Sir Run Run Shaw Hospital, School of Medicine, Zhejiang University, Hangzhou, P. R. China.

**Keywords:** papillary thyroid carcinoma, tandem-mass tag, markers, invasiveness, differentially expressed proteins

## Abstract

**Background and Aim:** Invasion and metastasis are critical events in papillary thyroid carcinoma (PTC) progression. Protein markers specific to this process may avoid over-treatment and urgently needed.

**Methods:** TMT-labeled mass spectrometry-based proteomics were carried out on PTC and invasive phenotype (iPTC) (3 pairs per group) and cross validate differentially expressed proteins (DEPs) (FC>1.5 and <0.67 and p<0.05) with GEO and TCGA datasets and the correlation genes of DEPs were also analyzed.

**Results:** We identified and quantified 4607 proteins identical to PTC and iPTC groups. Among which 12 DEPs in PTC and 179 DEPs in iPTCs were found. Cross-validation with GSE60542 and TCGA database revealed 10 DEPs that all significant correlated with metastasis and staging. Upregulated SLC27A6 showed negative correlation with 6 out of 9 downregulated DEPs including HGD, CA4, COL23A1, SLC26A7, FHL1 and TPO.

**Conclusion:** The panel of 7 genes (SLC27A6 and 6 downregulated DEPs) could have ideal prediction value to improve our understanding of invasiveness of PTC.

## Introduction

Thyroid cancer incidence has increased dramatically in many countries in the developed world over the past three decades 1. Papillary (thyroid) microcarcinoma (PTMC, or PMC) is the predominant variant of papillary thyroid cancer (which accounts for 85-90% of all thyroid cancers). According to clinical guidelines, a total lobectomy or subtotal/total thyroidectomy with radioactive iodine ablation is adequate and recommended for PTMC treatment 2. Increased diagnosis has led to concerns about over-diagnosis and over-treatment 3. Mortality from PTC remains low and stable. However, lymph node metastasis is a worse factor and cervical lymph node metastasis is more prevalent in PTC patients, with an incidence of 33.4% 4. Therefore, identifying tissue protein markers to predict early PTC invasiveness can allow timely intervention for the disease, and avoid over treatment in non-invasive nodules or PTC without metastasis.

Diagnosis of PTC nodules mainly depends on ultrasound and fine-needle aspiration (FNA) biopsy. Next-Generation Sequencing (NGS) is the most extensively applied molecular pathology method for detection of gene mutations and rearrangements such as BRAF, TERT, and RAS mutations, and rearrangements such as RET/PTC and ETV6-NTRK3 rearrangements 5, 6. However, the positive predictive value of sequencing is affected by the tissue heterogeneity in FNA sampling 7. Moreover, although methods to identify benign and malignant nodules through gene expression panels are reported, further validation is needed 8-10.Proteins play an essential role in bridging genotypes and phenotypes and execute their functions. Due to a variety of transcriptional and post-transcriptional modifications, gene expression levels may not be entirely consistent with protein levels 11, 12. Therefore, unveiling more precise protein expression patterns in thyroid cancer tissue to illustrate the potential transition from PTC to its invasive phenotypes is urgent and necessary.

Liquid chromatography-tandem mass spectrometry (LC-MS/MS) is an efficient empirical method for simultaneous identification and quantification of proteins in different samples 13. This high-throughput strategy has been applied in many diseases, including pulmonary disease 14, cerebrovascular disease 15, cardiac diseases 16, etc. The number of identified differentially expressed proteins (DEPs) in PTC compared to normal tissue, based-on LC-MS studies, has increased year by year thanks to the rapidly improving technologies. For example, in 2011, Yoshiyuki Ban et al. detected 524 proteins in PTC by shotgun LC-MS/MS 17. In 2016, Juan Martínez-Aguilar et al. found 1629 proteins by data-independent acquisition (SWATH)-MS analysis 18. In 2017, Zhang H et al. identified 2983 proteins by iTRAQ-coupled 2D LC-MS/MS 14. Finally, in 2018, a proteomics research based on high-resolution label-free mass spectrometry identified 2788 proteins 19. These proteomic studies using mass spectrometry have improved our knowledge of PTC and provided new insights into potential diagnostic markers discovery. However, the number of proteins identified should increase even further according to the progress of MS-based methods. Tandem-mass tag (TMT) technology is another powerful LC-MS/MS-based analytical method for sensitive, multiplexed, and identical peptide/protein quantification in tumor and its adjacent tissues, with the lowest system error. Although most studies focus only on the individual DEPs or gene markers in tumors compared with adjacent tissue, the correlation of these differentially expressed molecules may also reveal novel and critical networks involved in pathological states.

To unveil the DEPs that indicate invasive potential, we applied TMT-labeling to analyze 3 pairs of PTCs without and with intrathyroidal invasive papillary thyroid carcinoma (iPTC) and validated expression of candidates by western blot and GSE and The Cancer Genome Atlas (TCGA) datasets. We also analyzed the clinical significance and correlation of those genes. Our results provide novel insights into the biology of PTC invasion.

## Materials and Methods

### Clinical specimens

Under Medical Ethics and Human Clinical Trial Committee of Shanghai Tenth Hospital approval and informed consent were signed voluntarily, the inclusion criteria was female (thyroid cancer was one the most common cancers of female ) and clinically diagnosed with PTC, the exclusion criteria was patients with any basic disease like hypertension, diabetes or any disfunction of liver and kidney and so on. We collected thyroid cancer tissues immediately after surgery from Shanghai Tenth People's Hospital. Samples included 3 pairs of PTC and 3 pairs of intrathyroidal invasion (iPTC) and corresponding adjacent thyroid gland as a control. All tissues were reviewed by an endocrine pathologist to confirm the diagnosis. Table S1 lists the clinical information of patients in this study.

### Experimental procedures

#### Tissue lysis and protein extraction

**Figure 1:**
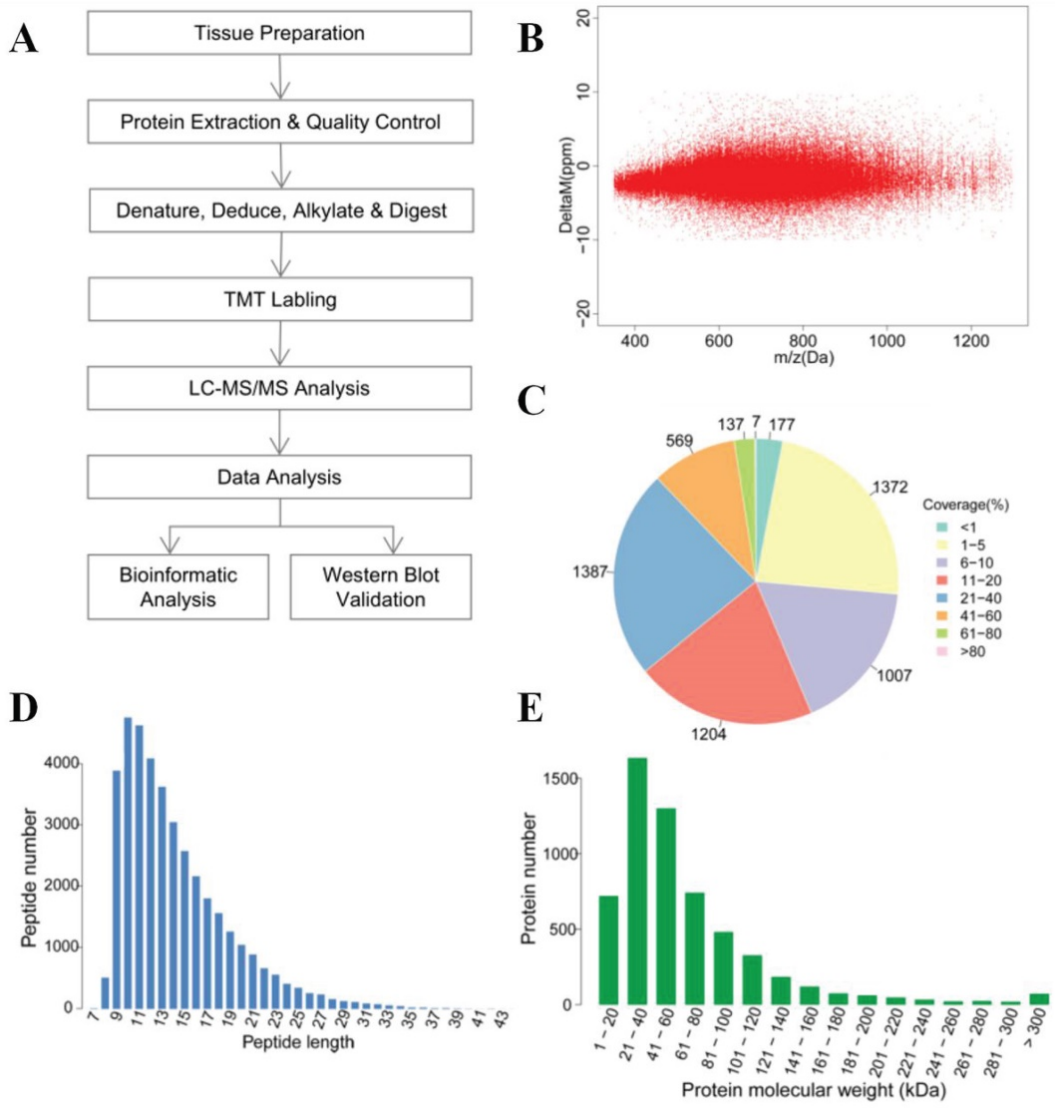
Overview of the proteomics analysis of thyroid carcinoma tissues. (A) General workflow for the liquid chromatography-tandem mass spectrometry (LC-MS/MS) analysis coupled with tandem-mass tag (TMT) reagents labeling. (B) Mass error distribution of identified peptides. (C) Coverage distribution of the identified proteins. (D) Length distribution of the peptides. (E) Molecular weight distribution of identified proteins.

The overall technological process is outlined in (Figure 1A). Protein detection and analysis were performed by Shanghai Majorbio Bio-pharm Technology Co., Ltd. All the samples were washed three times with cold PBS immediately after surgery and snap-frozen in liquid nitrogen. Samples were ground into powder in liquid nitrogen and extracted with Lysis buffer added at a ratio of 1:12 tissue weigh (mg): buffer volume (μl) (8M urea, 1% SDS, including protease inhibitors (Halt^TM^ Protease Inhibitor Cocktail 100×Prod # 78430, Thermo, IL. USA). Then, the samples were lysed on ice for 30 min, centrifuged for 20 min at 12000*g*, and protein supernatants were collected. BCA assay was carried out for measurement of protein concentration. Then, 100μg of each sample were used for MS analysis, and the same amount (mass) of each sample was mixed as an internal control. Ten millimolar TCEP (PG82080, Thermo, Rockford. USA) was added at 37°C for 1 h. Subsequently, 40 mM iodoacetamide (I6125, Sigma, USA) was added at 16 °C for 40 min. Cooled acetone was added to each tube (volume ratio=6:1), and the mixture was precipitated at 20°C for 4 h. Samples were centrifuged at 10,000*g* for 20 min, and the supernatant was discarded. The sample was then solubilized with 100μl Tetraethyl Ammonium Bromide TEAB (100 mM), and trypsin (V5280, Promega, USA) was added for enzymolysis (mass ratio = 1:50) overnight at 37°C. TMT 10-plex Isobaric Label Reagent (90111, Thermo, USA) was added to polypeptide samples at room temperature for 2 h (a tube of TMT per 100μg polypeptide), and then hydroxylamine was added for 15 min at room temperature. Table S1 also shows the sample parameters and TMT labeling information. The labeled polypeptide mixtures were collected and dried in a vacuum concentrator for further analysis.

#### LC-MS/MS analysis

The reverse-phase high pH liquid chromatography (RP-HPLC) separation was achieved on Waters ACQUITY UPLC (Waters, USA) equipped with C18 Column (1.7μm, 2.1 mm×150 mm, Waters, USA) at a flow rate of 200μl/min. A total of 20 fractions were collected, merged into 10 fractions. Liquid chromatography (LC) was performed by EASY-nLC 1200 (Thermo, USA) equipped with C18 column (75 μm×25 cm, Thermo Fisher, USA) at a flow rate of 300nL/min. The mass spectrometry was performed by Q-Exactive (Thermo, USA) with the following Dynamic Exclusion™ settings: the 20 most intense ions (m/z scan: 350-1,300, acquisition mode: DDA) were selected to be scanned. The HCD MS-1 was scanned with R=70,000, followed by MS-2 scans with R=35,000 (dynamic exclusion time: 18s). Raw data were collected by Thermo Xcalibur 4.0 (Thermo, USA). We used protein sequences with peptide mass errors lower than 10 ppm for database searching (Figure 1B). The analysis of MS raw data was performed by Proteome Discoverer^TM^ Software 2.1, and protein sequences were downloaded from UniProt (http://www.uniprot.org/proteomes/UP000005640) with a total number of 71591 sequences (download date: Sept 12th, 2017).

#### Validation of DEPs in iPTC by GSE and TCGA sources

To validate whether the DEPs were consistent at mRNA level and genomic level, we also analyzed GEO datasets using the GPL570 platform which included 11 PTC samples from N0 (no lymph node metastasis) and 13 from N+ (with lymph node metastasis) patients (GEO dataset: GSE60542) . At the genomic level, we analyzed TCGA dataset from UALCAN (http://ualcan.path.uab.edu/index.html) and GEPIA (http://gepia.cancer-pku.cn/) website platforms. We downloaded top up- and downregulated 250 genes from UALCAN and crossed the common genes using Bioinformatics & Evolutionary Genomics http://bioinformatics.psb.ugent.be/webtools/Venn. We further investigated the clinical significance by analyzing overall survival (OS) or disease-free survival (DFS) from GEPIA; UALCAN website also provided information regarding histological subtypes, individual cancer stages, and nodal metastasis. Positive and negative correlation (Pearson Correlation Co-efficient greater than 0.3) for each gene were downloaded, common genes were analyzed and networks were constructed on Excel. Hierarchical clustering was performed using HemI software.

#### Western blot analysis

Total proteins were extracted from both tumor and adjacent thyroid tissues of PTC and iPTC patients (Table 1). For each sample, 50μg protein was loaded into the wells of SDS-polyacrylamide gels (10%). Gels were run for separation at 80 V and then at 120V. Proteins were transferred onto polyvinylidene fluoride (PVDF) membranes by using the Bio-Rad Mini-Trans-Blot system. The membranes were then blocked with 5% fat-free milk in PBST (PBS and 0.1% Tween-20) for 2h at room temperature, followed by incubation overnight at 4°C with primary antibody dissolved in blocking buffer. The antibodies used were rabbit monoclonal anti-Versican (VCAN) antibody (dilution 1:1000, Abcam, ab177480), rabbit polyclonal anti-Cadherin16 antibody (dilution 1:400, Proteintech, 15107-1-AP), and rabbit-based anti-β tubulin antibody (dilution 1:1000, Cell signaling cycle, #2146). The secondary antibody was goat anti-rabbit IgG/HRP antibody (dilution 1:1000, Solarbio, SE134), dissolved in blocking buffer and incubated for 1 h at room temperature. Images were acquired by Amersham Imager 600 (GE, USA). Gray values were obtained using a fixed size rectangle feature enclosing the band of interest to obtain the intensity output (median intensity of pixels of the rectangle area) after background subtraction and contrast enhancement by Image J program.

#### Bioinformatics Analysis

Bioinformatics were performed using online available website (http://metascape.org/gp/index.html) to evaluate the DEPs, which included protein functional annotation, enrichment analysis, and protein-protein interaction analysis. Online gProfiler(http://biit.cs.ut.ee/gprofiler/gost) were also used for gene function classify.

#### Statistical analysis

The statistical analysis of LC-MS/MS data was performed by R software 3.4.3. We used protein abundance ratio as statistical data. DEPs in pairwise comparisons were identified by two-sided t-test. The cut-off of DEPs is set at 1.5-fold change and p < 0.05. For western blot assay, triplicate was carried out in PTC and iPTC vs adjacent tissue. Quantification of band were scanned and analyzed by Image J software and t-Test and set the criteria at FC>0.5, p<0.05 and FDR<0.05 for significant differences. Heat maps were generated by hierarchical clustering by HemI software. Univariate survival analysis and multivariate analyses were carried out using the Kaplan-Meier Method based on online tools. We chose p < 0.05 as the level of significance.

## Results

### Quantitative analysis of proteins by TMT labeling LC-MS/MS

To systematically analyze the protein profiles of PTC and iPTC, we performed MS analysis (Figure 1A). Table S2 lists the general quantitative information of the identified proteins. We identified 134738 spectra in total, and got the quantitative information of 5860 proteins. Among these, 5221 proteins were mapped in tumor and non-tumor tissues of PTC, and 4607 proteins were overlapping in both PTC and iPTC (Table 1, Table S2 and S3). Among the identified peptides, the majority of mass errors were distributed in -5 ppm to 0 ppm (Figure 1B). The coverage of most proteins ranged from 1% to 40%; the number of proteins with a coverage greater than 10% accounted for 56.4% of 5860 (Figure 1C). These results showed an acceptable mass accuracy of the MS data. The length of the majority of the peptides ranged from 8 to 23 amino acids (Figure 1D) which confirmed the complete digestion of the proteins in our sample preparation. Proteins with a molecular weight greater than 100kDa accounted for 16.8% (Figure 1E), providing abundant information for analysis of macromolecular proteins. These results demonstrate the credibility of the protein profile provided by the TMT-labeled mass spectrometer.

### Marked DEPs in PTC and iPTC based on TMT analysis

**Figure 2:**
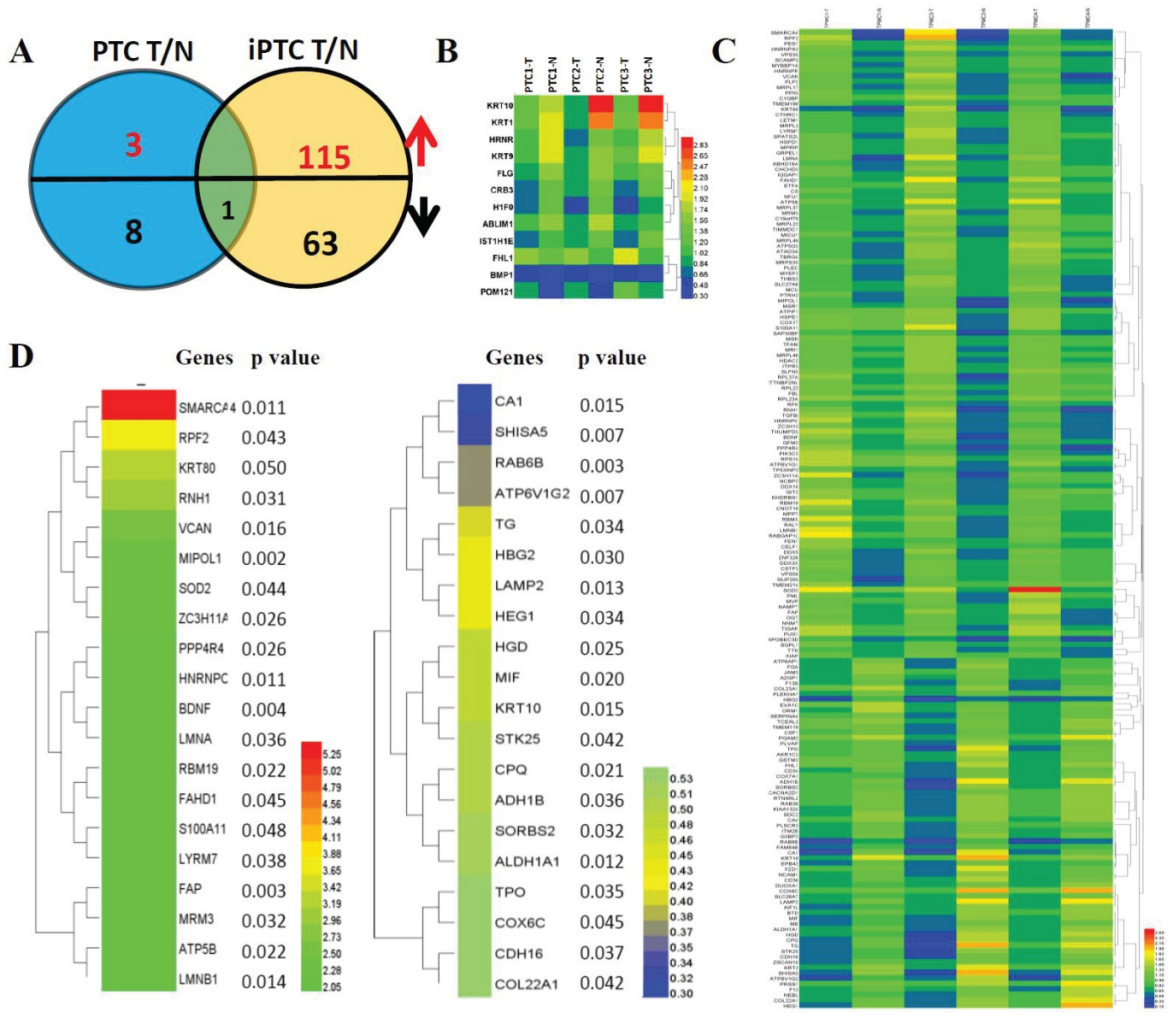
Hierarchical cluster analysis of differentially expressed proteins (DEPs) in paired thyroid cancers with their corresponding adjacent normal tissues. (A) Significant differentially expressed proteins (DEPs) in papillary thyroid carcinoma (PTC) and invasive papillary thyroid carcinoma (iPTC) groups (Fold change ≥ 1.5; P < 0.05). (B) Hierarchical clustering for 12 DEPs (Fold change ≥ 1.5; P < 0.05) in PTC tumor compared with its adjacent tissues (n=3) using MEV4.7.1 software. (C) Hierarchical clustering for 179 DEPs (Fold change ≥ 1.5; P < 0.05) in iPTC tumor compared with its adjacent tissues (n=3) using MEV4.7.1 software. (D) Hierarchical clustering of top 20 up(left)- and down(right)-regulated DEPs in iPTC group.

Hierarchical cluster analysis of protein expression showed a clearly different expression profile between tumor tissues and adjacent thyroid tissues in both PTC and iPTC. We used t-Test and set the criteria at FC>0.5, p<0.05 and FDR<0.05 for significant differences in expression of identified proteins in PTC and iPTC vs adjacent tissue. By comparison of tumor versus normal tissue (mentioned thereafter for T and N, respectively) in PTC, 3 protein increased and 9 proteins decreased (0.06% and 0.23% of 4607, respectively; (Figure 2A and 2B, Table S4). In iPTCs, 179 DEPs included 115 upregulated and 64 downregulated proteins (1.95% and 1.32% of 4607), respectively; (Figure 2C and Table S5), Furthermore, by comparing common proteins of iPTC/PTC, we found only 1 protein, KRT10, significantly downregulated in tumor tissues (0.46% and 1.98% of 4607, respectively), The top 20 up- and downregulated genes in iPTC are listed in (Figure 2D). we also analyzed the differential expressed proteins between thyroid cancer(combined PTC and iPTC) and iPTC/PTC groups for finding the in situ and invasion process specific proteins, we found 8 common genes(0.173% of 4607) including 5 down and 3 up-regulated proteins between two groups. In tumorigenesis of PTC, 29 proteins(0.63% of 4607)were enriched, and in iPTC/PTC, 90 proteins (1.95% of 4607)were also found specific to invasion process (Fig S1 and Table S6).

### Bioinformatic analysis for DEPs in PTC and iPTC

**Figure 3:**
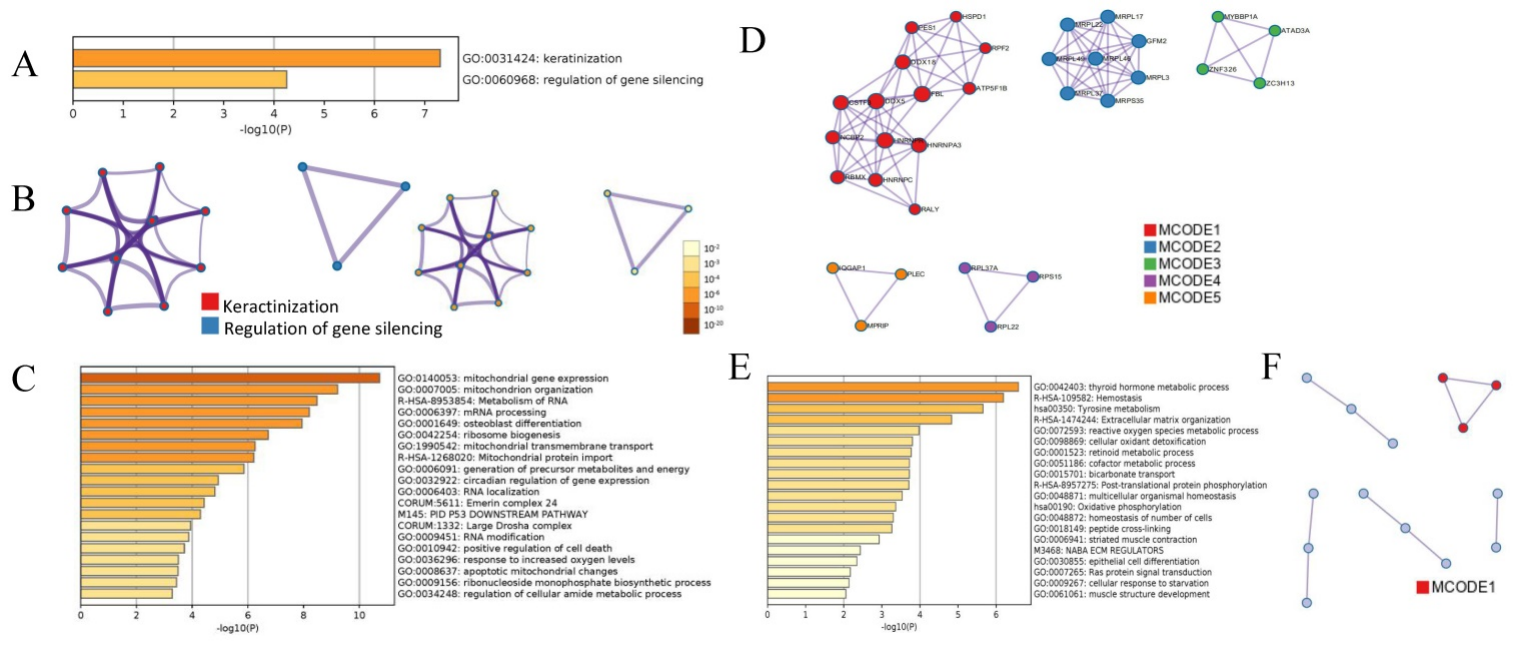
Bioinformatic analysis of DEPs in PTC and iPTC using online metascape (http://metascape.org/gp/index.html). (A)Bar graph of enriched GO across PTC, colored by p-values. (B)Protein-protein Interaction (PPI) Enrichment Analysis for 12 proteins in PTC group and colored by colored by cluster ID(left) and by p-value(right). (C) Bar graph of enriched. GO across 115 upregulated DEPs in iPTC and colored by p-values; (D) PPI network and the Molecular Complex Detection (MCODE) components were identified. (E) GO process for 64 downregulated DEPs in iPTC group; (F) PPI network and MCODE components were identified in 64 downregulated DEPs in iPTC.

Bioinformatics analysis showed that 12 DEPs in PTC were enriched in 2 major gene ontology (GO) biological processes, keratinization and regulation of gene silencing (Figure 3A and 3B), indicating that the altered biological processes in PTC are simpler than those in iPTC group. Of the 179 DEPs identified in iPTC, the 115 upregulated DEPs were classified into 29 clusters. The top 5 enriched GO biological processes (BP) based on Log10(p) included mitochondrial gene expression (12/115, 10.43%), mitochondrion organization (17/115, 14.78%), metabolism of RNA (18/115, 15.65%), mRNA processing (16/115, 13.91%), and osteoblast differentiation (11/115, 9.56%) (Figure 3C). Protein protein-interaction (PPI) of Molecular Complex Detection (MCODE) showed that 115 upregulated genes comprised 5 MCODE networks (Figure 3D), and the major MCODEs were 1, 2, and 4. MCODE1 consisted of 14 proteins, which is the top number of proteins among the 5 MCODEs that included 3 pathways: CORUM:1181 (C complex spliceosome), R-HAS-72163 (mRNA splicing-major pathway), and R-HAS-5419276 (mRNA splicing). MCODE2 ranked the top based on Log10(p), and included 3 mitochondrial processes, 1 mitochondrial translation elongation and 2 termination processes. Regarding the 64 downregulated genes, GO analysis showed the top 5 GO biological processes included thyroid hormone metabolic process (4/64, 6.25%), hemostasis (17/64, 26.56%), extracellular matrix organization (7/64, 10.94%), reactive oxygen species metabolic process (6/64, 9.38%), and cellular oxidant detoxification (4/64, 6.25%). The two major KEGG pathways were Tyrosine metabolism (4/64, 6.25%) and Oxidative phosphorylation (4/64, 6.25%). PPI Enrichment analysis found that only one MCODE was enriched, namely hsa00190: Oxidative phosphorylation (Figure 3E and 3F).

### Validation of MS data by TCGA and GEO datasets and western blot

**Figure 4:**
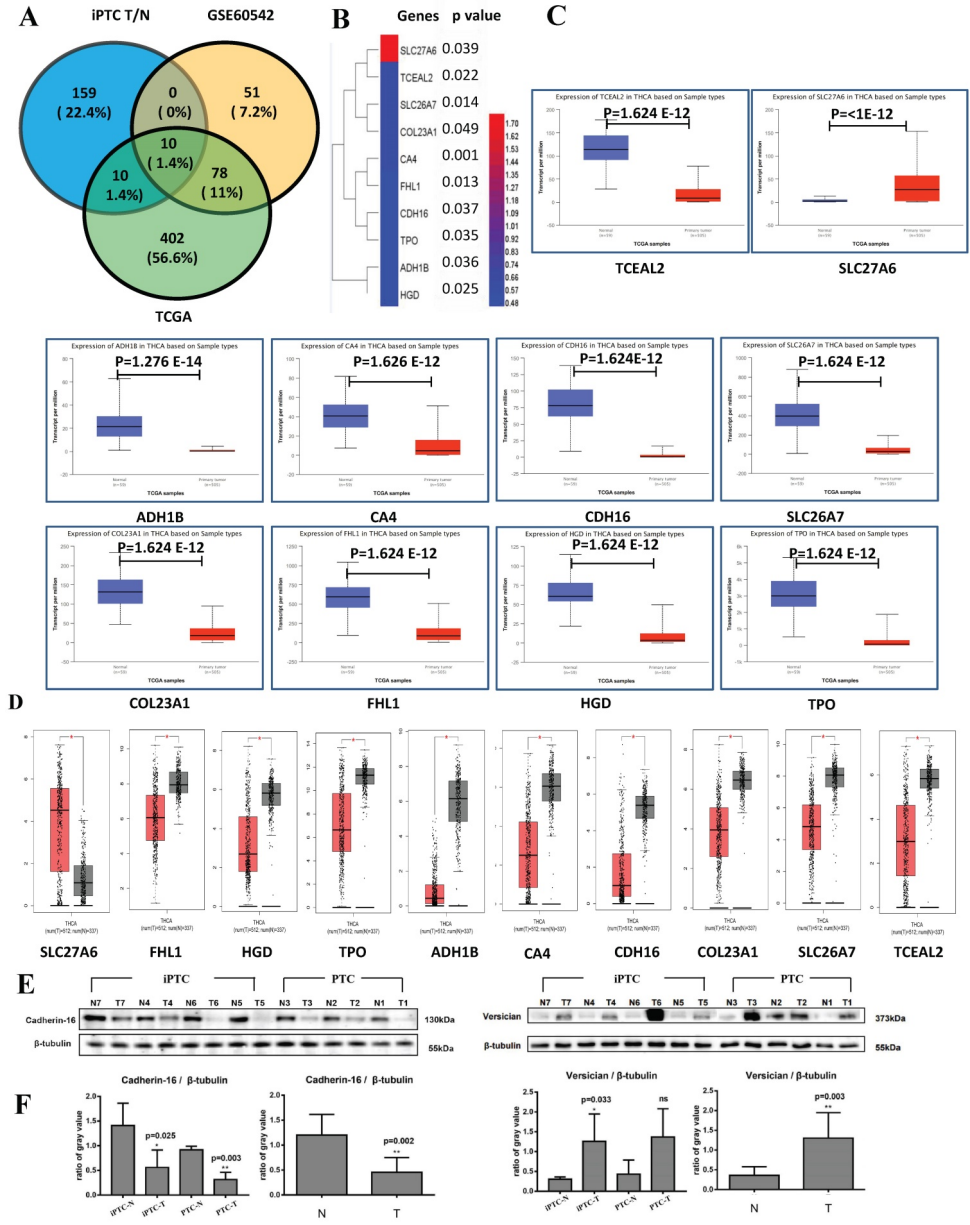
Validation of DEPs by TCGA datasets and western blot. TCGA data were accessed through GEPIA (N=337 and T=512) and UALCAN platform (N=59 and T=505). (A) The Venn diagrams of DEPs crossed by iPTC/N, GSE60542 (FC>4, p-value<0.05, FDR<0.05) and UALCAN website (Top 250 up- and downregulated genes) show 10 proteins including 1 upregulated (SLC27A6) and 9 downregulated (TCEAL2, SLC26A7, COL23A1, CA4, FHL1, CDH16, TPO, ADH1B, HGD). (B) Hierarchical clustering for 10 crossed DEPs (Fold change ≥ 1.5; P < 0.05) using HemI software. (C-D) Validation of 10 crossed proteins by GEPIA and UALCAN website. (E-F) Validation of DEPs using western blot on the same samples subjected to LC-MS detection. CDH-16 and versican (VCAN), that were down and upregulated proteins in figure, were chosen for the validation, and intensity results were quantified by Image J program. proteins expression was normalized to β-tublin. Error bars are the standard error of the mean and statistical significance P < 0.05 and 0.01 are noted using * and **, respectively.

To verify our results at the mRNA and genomic level and validate those proteins that are expressed consistently, we also analyzed GSE60542, which contains 13 N+(indicate with lymph node metastasis) and 11 N0(indicate without lymph node metastasis) PTC samples with the criteria FC>4 or <0.25 and p<0.05, and TCGA datasets from UALCAN platform (N=59, T=505 N represent Normal and T represent Tumor), which contain top 250 up- and downregulated genes from the section of "Thyroid carcinoma" (Table S7). We found 10 common genes, of which 1 upregulated (SLC27A6) and 9 downregulated (TPO, HGD, FHL1, SLC26A7, COL23A1, CDH16, CA4, TCEAL2, and ADH1B) (Figure 4A and 4B). For further verification, we input the above genes in two TCGA platforms, GEPIA (N=337 and T=512) and UALCAN. All 10 genes showed significantly different expression consistent results in both websites (Figure 4C and 4D) that confirm the reliability of our MS results. Then, western blot was conducted in 4 pairs of iPTC, as well as 3 pairs of PTC sample. We chose CDH16, one downregulated DEPs, and VCAN, up regulated in our MS results and out of the 10 DEPs and elevated in PTC but not significant (average FC=1.40), and protein expression for both confirmed our MS results (Figure 4E and 4F). These results suggested that the 10 DEP panel showed reliable consistency at the genomic, transcript, and protein levels in iPTC.

### Analysis of staging and metastasis of DEPs

Invasion and metastasis are lethal clinical parameters and inversely correlated with cancer patients' survival. To analyze their clinical significance, we examined the association of the 10 DEPs with staging and metastasis on UALCAN. Results showed that all 10 proteins were notably correlated with clinical staging and metastasis (Fig. S2). OS and DFS are of great importance for cancer patients. Thus, we further analyzed the OS in relation with the 10-DEP panel using both GEPIA and UALCAN. Of these, *FHL1, TPO, COL23A1, HGD,* and *SLC26A7* in UALCAN website were significantly associated with patients' survival. Moreover, *TPO, ADH1B* and *FHL1* were significantly associated with survival on GEPIA website. *TPO* and *FHL1* exhibited the same clinical significance on both websites (Fig. S3), suggesting they play a more important role in PTC than the other 8 genes.

### Correlation analysis of 10 DEGs found 7 DEPs panel

**Figure 5:**
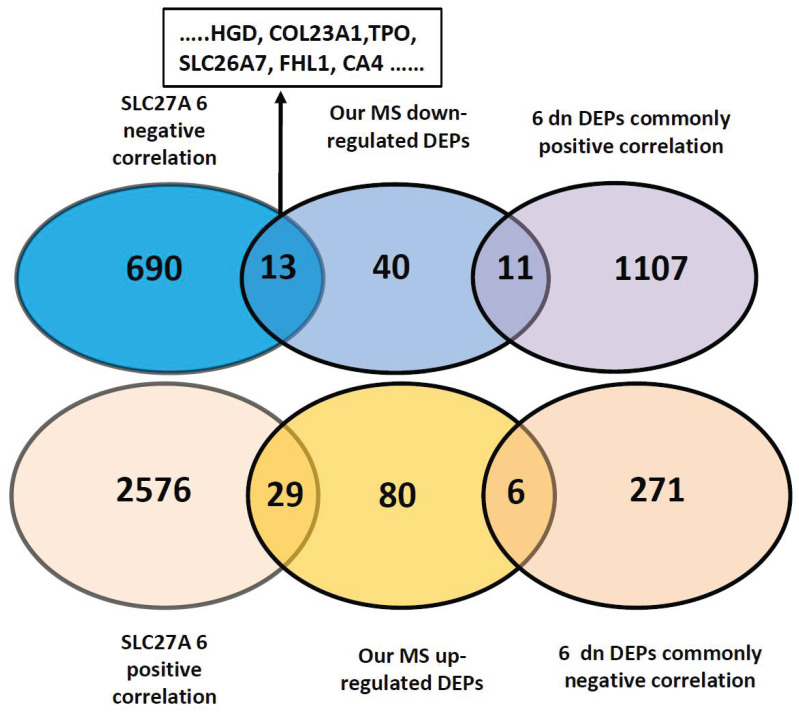
Correlation analysis of 7 DEPs with primary findings and other genes of thyroid cancer. Correlating genes (Pearson CC >0.3) were accessed from UALCAN website platform. The negative correlation of upregulated SLC27A6 with 6 downregulated DEPs (HGD, COL23A1, TPO, SLC26A7, FHL1, CA4) out of 9 DEPs were predicted by TCGA database. Genes positively and negatively correlated with SLC27A6 in thyroid cancer were crossed with our MS results (115 up and 64 downregulated DEPs) and 2605 positively and 703 negatively correlated genes were found. Among these, 29 proteins were crossed with our 115 upregulated DEPs, and 13 with our 64 downregulated DEPs. Common genes positively and negatively correlated with the 6 downregulated DEPs were 277 and 1118, respectively. Among these, 6 and 11 genes were overlapping with 115 up- and 64 downregulated DEPs, respectively.

Tumorigenesis is a complex process that encompasses multiple stages and mechanisms. Alteration of gene panels reflects pathogenesis far more comprehensively than that of single genes. To find the underlying correlation of DEGs with invasiveness/metastasis, we downloaded the genes positively and negatively correlated with the 10 DEPs from UALCAN and analyzed their correlations. We first analyzed the only upregulated SLC27A6, and revealed 703 and 2605 negatively and positively correlated genes in thyroid cancers (Pearson CC greater than 0.3), respectively (Figure 5 and Table S7). By comparison with 179 DEPs found in our primary results, we found common genes of 13 of 64 downregulated and 29 of 115 upregulated DEPs and DEPs. Among the13 DEPs, 6 genes (*HGD, COL23A1, TPO, SLC26A7, FHL1* and *CA4*) that are comprised within the 9 downregulated DEPs identified by database validation (Table 2). So, we next using 6 downregulated DEPs that negatively correlated with SLC27A6 for further analysis. We found 1118 common genes positively correlated with 6 DEPs, that contains 11 common DEPs out of our 64 downregulated DEPs. The common genes negatively correlated with 6 downregulated-DEPs were 277, including *MVP* and *S100A11, PLP2, SPATS2L, KRT80 and SLC27A6* (Table 3, Table S8,S9 and S10).

Interestingly, we analyzed the 9 downregulated DEPs, except for *ADH1B*, 8 genes showed good correlation with one another, in which, *TPO, HGD,* and* FHL1* as top 3 ranking in these 8 genes in Pearson CC score (Table 2). Analysis of the positively and negatively correlated genes by GO and KEGG revealed that top enriched GO terms were organic anion transport and mitochondria-related process suggesting that energy metabolism (Figure 3C) and organic anion balance are the predominant factors for initiation of PTC invasion. These results strongly indicate that the 7 DEPs penal are closely correlated with and could be the core genes contributing to the invasive potential.

## Discussion

Over-diagnosis and -treatment of thyroid cancer are gaining attention due to the rapidly increased occurrence worldwide 20-22. Protein markers that predict the invasion and metastasis potential could serve as meaningful factors to improve thyroid cancer treatment. In this study, we found 10 DEPs from iPTC tissues that correlated with metastasis and staging. Among these, SLC27A6 was upregulated and showed negative correlation with 6 out of 10 DEPs. These 6 downregulated genes also showed positive correlation with one another. This 7 DEPs panel could allow to predict invasiveness and provide new insights into the biology of iPTC.

Based on our LC-MS/MS results, in total, we identified 12 DEPs in PTC and 179 in iPTC groups compared with corresponding controls. Only one protein, KRT10, an intermediate filament (IF) family member, was downregulated in both PTC and iPTC samples. By combined 6 tumors(including PTC and iPTC) versus adjacent tissue. We have found 8 common genes including 5 genes downregulated and 3 genes upregulated (Fig. S1).29 genes were correlated with tumoirgenesis of thyroid cancer and 90 genes were specific to invasion. KRT10 was reported to be correlated with the stability of skin structure and is often mutated in ichthyosis with confetti, a skin disorder 23. Downregulation of KRT10 indicates its pathological roles in invasion initiation and in PTC pathology.

Increasing evidence suggests that reprogramming energy metabolism provokes invasion and metastasis. In our analysis, the 179 DEPs we identified encompass 115 up- and 64 downregulated proteins. The major biological processes that include these proteins are mitochondrial-associated processes, mRNA splicing or processing, instability of hemostasis and extracellular structure, and thyroid hormone metabolic process. The top upregulated biological processes here were related to mitochondrial energy metabolism, one of the important hallmarks of cancer 24, and the top downregulated biological processes are associated with thyroid hormone metabolic process. Taken together, these results highlight the impact of energy and thyroid hormone metabolism on the invasiveness and progression of PTC.

By online database validation, we identified a panel of 10 proteins playing an essential role in iPTC. Using online sources, we found that all 10 proteins were closely correlated with PTC staging and metastasis, underlining their meaningful role in PTC. Among these, SLC27A6 is a novel protein and the only upregulated protein identified that functions as a transporter mediating long-chain fatty acid uptake 25. SLC27A6 was a specific upregulated gene to invasion process by analysis of iPTC/PTC in our study(Table S6). Previously, SLC27A6 was only reported to be downregulated in breast cancer, suggesting that the role of this protein may change in different tumor environments. Our findings illustrate the potential of SLC27A6 as a marker for PTC invasiveness.

Among the 9 downregulated proteins, 5 proteins were consistent with the literature reported, namely SLC26A7 26, CA4 27-29, FHL1 30, CDH16 31, and TPO 32-34. Four proteins, including TCEAL2, COL23A1, ADH1B 35, and HGD were novel proteins identified as differentially expressed in iPTC. Literature shows a pathological role of TCEAL2 only in ovarian carcinoma 36 and of COL23A1 only in clear cell renal cell carcinoma (ccRCC) 37. ADH1B was found to be correlated with alcohol consumption-related cancer 36, while HGD was reported in alkaptonuria 38 and possesses at least 11 mutations and variants 39 in TCGA database. In UALCAN platform, these 4 novel genes all showed significant correlation with staging and metastasis, strongly indicating that they are key players in the process of invasion.

Networks compromised by gene correlation play a central role in physiological and pathological process. Analysis of the correlation of 10 DEPs revealed 2605 and 703 positively and negatively correlated genes in thyroid cancers (Pearson CC greater than 0.3), respectively. By comparison with our primary findings, we found 29 of 115 upregulated DEPs and 13 of 64 downregulated DEPs. The 13 DEPs contain 6 genes (*HGD, COL23A1, TPO, SLC26A7, FHL1* and *CA4*) that are comprised within the 9 downregulated DEPs of the panel identified by database validation.

Interestingly, 8 of the 9 downregulated DEPs, namely TPO, HGD, FHL1, SLC26A7, COL23A1, CDH16, CA4, and TCEAL2 showed close correlation with one another (Table 2). Further, 1118 common genes positively correlated with the 6 downregulated genes that negatively correlated with SLC27A6. These included 11 downregulated proteins consistent with our LC-MS results (PLVAP, TCEAL2, NCAM1, CDH16, COX6C, ALDH1A1, SORBS2, STK25, KRT10, TG, and ATP6V1G2). Moreover, 277 common negatively correlated genes were found that contained 6 MS-based, upregulated proteins (KRT80, S100A11, PLP2, SPATS2L, SLC27A6, and MVP). Taken together, these results strongly suggested that those 7 genes panel play a central role in the pathological invasive process.

In terms of the overall survival, the critical parameter for prognosis, TPO and FHL1 were identified as the two significant common genes in the GEPIA and UALCAN platforms, indicating their potential value for prediction of patient prognosis. The limitation of this study was the limited sample number and single sex. We need to expand the sample number and gender for more comprehensive study. And different metastatic stage of thyroid cancer sample also needs to be enrolled for further investigation.

In conclusion, our current study has found a panel of 10 DEPs that could predict the invasiveness of PTC at early stage of tumors, and four novel proteins TCEAL2, COL23A1, ADH1B, and HGD differentially expressed in PTC. Among the 10 DEPs, the 7-gene panel (SLC27A6 and the 6 downregulated DEPs) could serve as core protein panel to predict the invasiveness of PTC. Our findings contribute new insights for understanding the invasive process and shed novel light on the management of thyroid cancer.

## Supplementary Material

Supplementary figures.

Supplementary table 1.

Supplementary table 2.

Supplementary table 3.

Supplementary table 4.

Supplementary table 5.

Supplementary table 6.

Supplementary table 7.

Supplementary table 8.

Supplementary table 9.

Supplementary table 10.

## Figures and Tables

**Table 1:** Identification information of quantitative qnalysis by TMT-labled LC-MS/MS

Total Spectra	Identified Spectrum	Peptide number	Protein number	Protein Number	Protein Number	Overlapping in PTC and iPTC
				in PTC	in iPTC	
592290	134738	39188	5860	5221	4607	4607

**Table 2:** The positive and negative correlation of 8 differential expressed genes with each other.

	TPO	HGD	FHL1	SLC26A7	COL23A1	CDH16	CA4	TCEAL2	Average
TPO	/	0.82	0.72	0.59	0.62	0.49	0.46	0.36	0.617
HGD	0.82	/	0.76	0.61	0.58	0.49	0.42	0.31	0.613
FHL1	0.72	0.76	/	0.66	0.55	0.43	0.42	0.36	0.59
SLC26A7	0.59	0.61	0.66	/	0.63	0.4	0.42	0.42	0.552
COL23A1	0.62	0.58	0.55	0.63	/	0.46	0.34	0.35	0.53
CDH16	0.49	0.49	0.43	0.4	0.46	/	0.31	NA	0.43
CA4	0.46	0.42	0.42	0.42	0.34	0.31	/	0.34	0.395
TCEAL2	0.36	0.31	0.36	0.42	0.35	NA	0.34	/	0.357
**SLC27A6^#^**	**-0.36**	**-0.38**	**-0.33**	**-0.34**	**-0.38**	NA	**-0.3**	NA	/

**^#^**Means that SLC27A6 showed negative correlation with 6 genes

**Table 3:** The list of crossed genes by correlation analysis.

	upregulated	downregulated
	115 DEPs	64 DEPs
SLC27A6 positive correlation	IQGAP CTTNBP2NL SPATS2L KRT80 MVP MSN PLEC PIK3C3 SGPL1 LMNA HDAC2 DDX18 KHDRBS1 HNRNPC VPS35 NAMPT BDNF HNRNPR ZNF326 XIAP SLFN5 S100A11 RBMX ZC3H13 ITPR3 MIPOL MYEF2 MPRIP SMARCA4 (***29 genes in total***)	/
SLC27A6 negative correlation	/	COX6C **HGD COL23A1** TG **TPO** KRT10 NCAM1 **SLC26A7 FHL1** SORBS2 COX7A1 **CA4** GSTM2 ***(13 genes in total)***
6 downregulated DEP negative correlation	MVP S100A11 PLP2 SPATS2L	/
	KRT80 SLC27A6 ***(6 genes in total)***	
6 downregulated DEP positive correlation	/	PLVAP TCEAL2 NCAM1 CDH16 COX6C ALDH1A1 SORBS2 STK25 KRT10 TG ATP6V1G2*** (11 genes in total)***
