# Supplementary material for: TMT-labeling Proteomics of Papillary Thyroid Carcinoma Reveal Invasive Biomarkers
Source: J Cancer. 2020 Aug 25;11(20):6122–32. doi: 10.7150/jca.47290 (PMC7477402; doi:10.7150/jca.47290)
Supplement: Supplementary file 1 — Supplementary figures. [file jcav11p6122s1.pdf]

**Figure S1**. Differential expressed genes(DEPs) tumorigenesis (in situ) and invasion process. A. analysis of DEPs from tumor (iPTC and PTC) and invasion process and found 8 common genes. specific DEPs in situ and invasion process were 29 and 90, respectively. B-C. 3 upregulated (B) and 5 downregulated (C) genes common to in situ and invasion process. *and ** represent p-value$<$0.01 and 0.05. respectively. D.GO and KEGG analysis of DEPs in iPTC/PTC process were carried out by only gProfiler.

**Figure S2**. Five genes including FHL1, TPO, SLC26A7, HGD and COL23A1 showed significant OS on THCA from UALCAN website(http://ualcan.path.uab.edu/index.html)

**Figure S3**. Three genes including FHL1, TPO and ADH1B showed significant OS on THCA from GEPIA website (http://gepia.cancer-pku.cn/)

**Figure S4.** Association of 10 DEPs with clinical staging and metastasis.
TCGA database indicated the nodal metastasis (left) and staging (right) for up- and downregulated genes in thyroid tissues compared to the normal tissues. Staging dataset contains normal tissues (n=59), N0 (n=230) and N1 (n=58). Nodal metastasis data including normal tissue (n=59), stage 1 (n=284), stage 2 (n=52), stage 3 (n=112) and stage 4 (n=55). P values were calculated by online tools.

# Fig S1

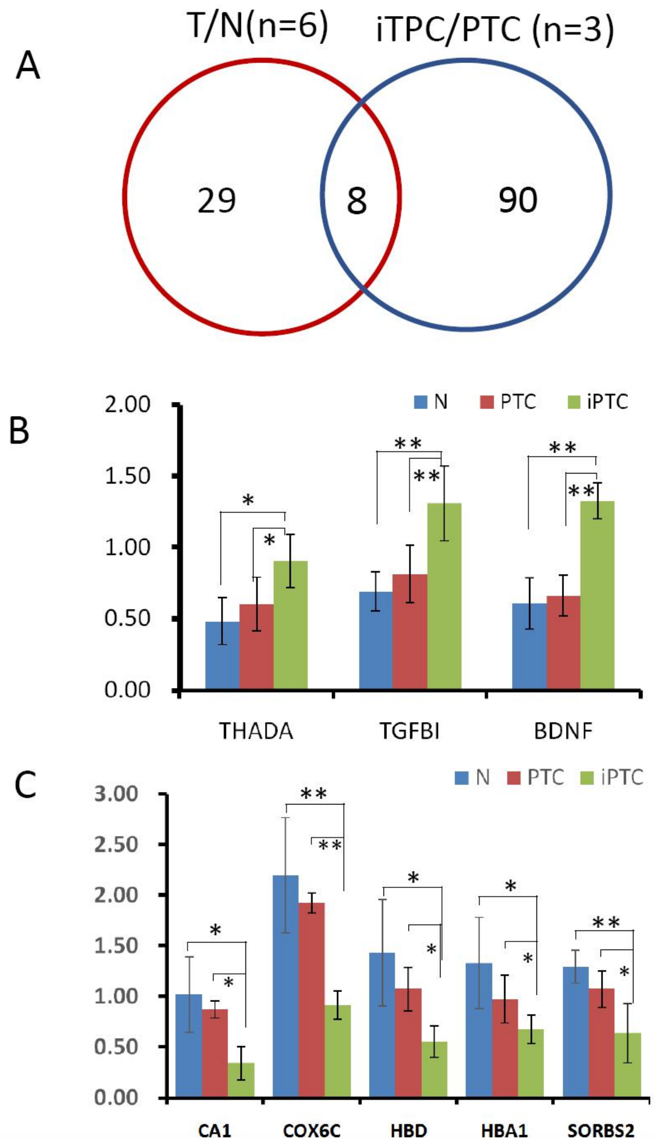


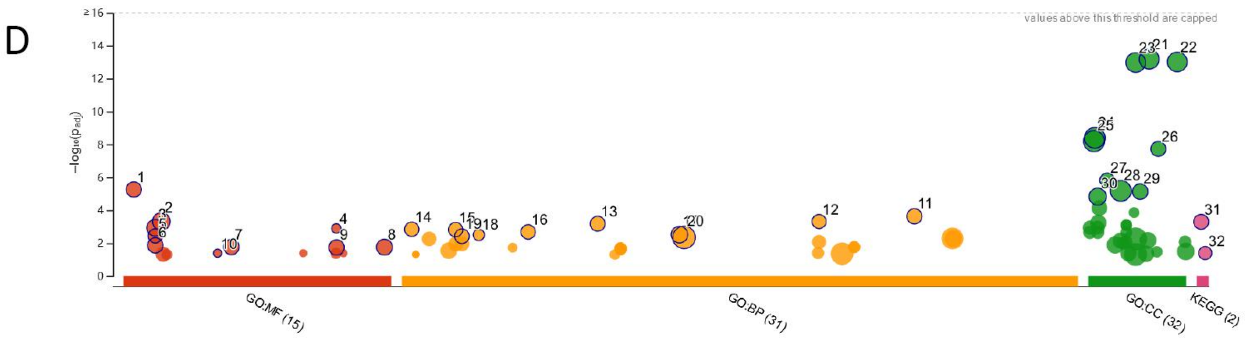


| ID | Source | Term ... | Term Name | $p_{adj}$ (query_1) |
|---|---|---|---|---|
| 1 | GO:MF | GO:0003735 | structural constituent of ribosome | $5.527\times10^{-6}$ |
| 2 | GO:MF | GO:0005198 | structural molecule activity | $4.797\times10^{-4}$ |
| 3 | GO:MF | GO:0004857 | enzyme inhibitor activity | $1.176\times10^{-3}$ |
| 4 | GO:MF | GO:0060228 | phosphatidylcholine-sterol O-acyltransferase activ... | $1.273\times10^{-3}$ |
| 5 | GO:MF | GO:0004867 | serine-type endopeptidase inhibitor activity | $3.592\times10^{-3}$ |
| 6 | GO:MF | GO:0004866 | endopeptidase inhibitor activity | $1.371\times10^{-2}$ |
| 7 | GO:MF | GO:0030414 | peptidase inhibitor activity | $1.699\times10^{-2}$ |
| 8 | GO:MF | GO:1901681 | sulfur compound binding | $1.754\times10^{-2}$ |
| 9 | GO:MF | GO:0061135 | endopeptidase regulator activity | $1.822\times10^{-2}$ |
| 10 | GO:MF | GO:0018636 | phenanthrene 9,10-monooxygenase activity | $4.103\times10^{-2}$ |
| 11 | GO:BP | GO:0140053 | mitochondrial gene expression | $2.342\times10^{-4}$ |
| 12 | GO:BP | GO:0070126 | mitochondrial translational termination | $4.811\times10^{-4}$ |
| 13 | GO:BP | GO:0032543 | mitochondrial translation | $6.619\times10^{-4}$ |
| 14 | GO:BP | GO:0001523 | retinoid metabolic process | $1.416\times10^{-3}$ |
| 15 | GO:BP | GO:0006415 | translational termination | $1.512\times10^{-3}$ |
| 16 | GO:BP | GO:0016101 | diterpenoid metabolic process | $2.080\times10^{-3}$ |
| 17 | GO:BP | GO:0043687 | post-translational protein modification | $3.008\times10^{-3}$ |
| 18 | GO:BP | GO:0007597 | blood coagulation, intrinsic pathway | $3.146\times10^{-3}$ |
| 19 | GO:BP | GO:0006721 | terpenoid metabolic process | $3.761\times10^{-3}$ |
| 20 | GO:BP | GO:0044237 | cellular metabolic process | $4.477\times10^{-3}$ |
| 21 | GO:CC | GO:0070062 | extracellular exosome | $6.612\times10^{-14}$ |
| 22 | GO:CC | GO:1903561 | extracellular vesicle | $9.644\times10^{-14}$ |
| 23 | GO:CC | GO:0043230 | extracellular organelle | $1.046\times10^{-13}$ |
| 24 | GO:CC | GO:0005615 | extracellular space | $3.996\times10^{-9}$ |
| 25 | GO:CC | GO:0005576 | extracellular region | $6.347\times10^{-9}$ |
| 26 | GO:CC | GO:0072562 | blood microparticle | $1.888\times10^{-8}$ |
| 27 | GO:CC | GO:0015934 | large ribosomal subunit | $1.502\times10^{-6}$ |
| 28 | GO:CC | GO:0031982 | vesicle | $6.675\times10^{-6}$ |
| 29 | GO:CC | GO:0044391 | ribosomal subunit | $7.406\times10^{-6}$ |
| 30 | GO:CC | GO:0005759 | mitochondrial matrix | $1.473\times10^{-5}$ |
| 31 | KEGG | KEGG:03010 | Ribosome | $5.026\times10^{-4}$ |
| 32 | KEGG | KEGG:04979 | Cholesterol metabolism | $4.016\times10^{-2}$ |

Fig S2

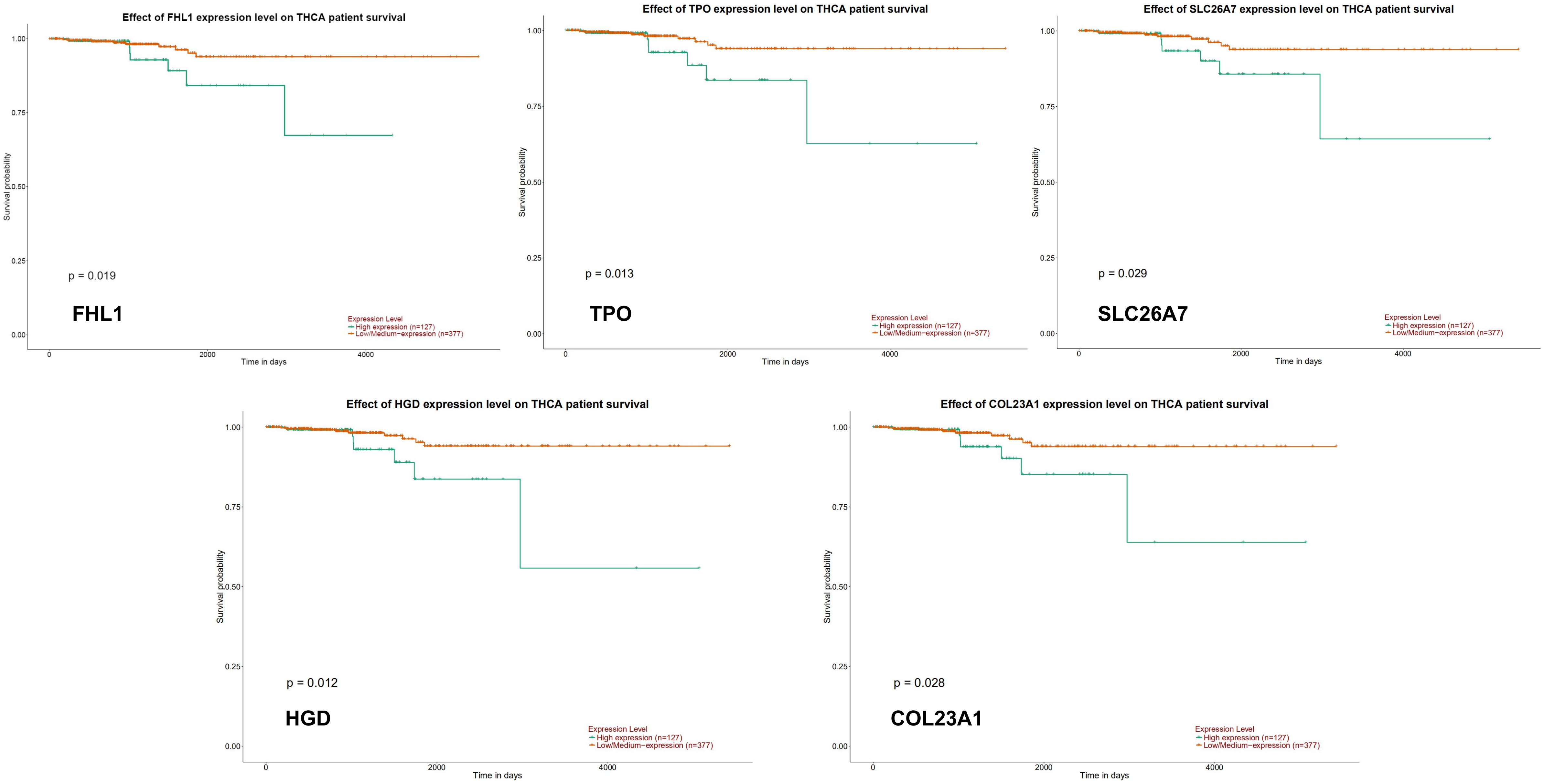

Effect of FHL1 expression level on THCA patient survival
Survival probability
1.00
0.75
0.50
0.25
0.00
0
2000
4000
Time in days
p = 0.019
FHL1
Expression Level
High expression (n=127)
Low/Medium−expression (n=377)
Effect of TPO expression level on THCA patient survival
Survival probability
p = 0.013
TPO
Expression Level
High expression (n=127)
Low/Medium−expression (n=377)
Effect of SLC26A7 expression level on THCA patient survival
Survival probability
p = 0.029
SLC26A7
Expression Level
High expression (n=127)
Low/Medium−expression (n=377)
Effect of HGD expression level on THCA patient survival
Survival probability
p = 0.012
HGD
Expression Level
High expression (n=127)
Low/Medium−expression (n=377)
Effect of COL23A1 expression level on THCA patient survival
Survival probability
p = 0.028
COL23A1
Expression Level
High expression (n=127)
Low/Medium−expression (n=377)

Fig S3

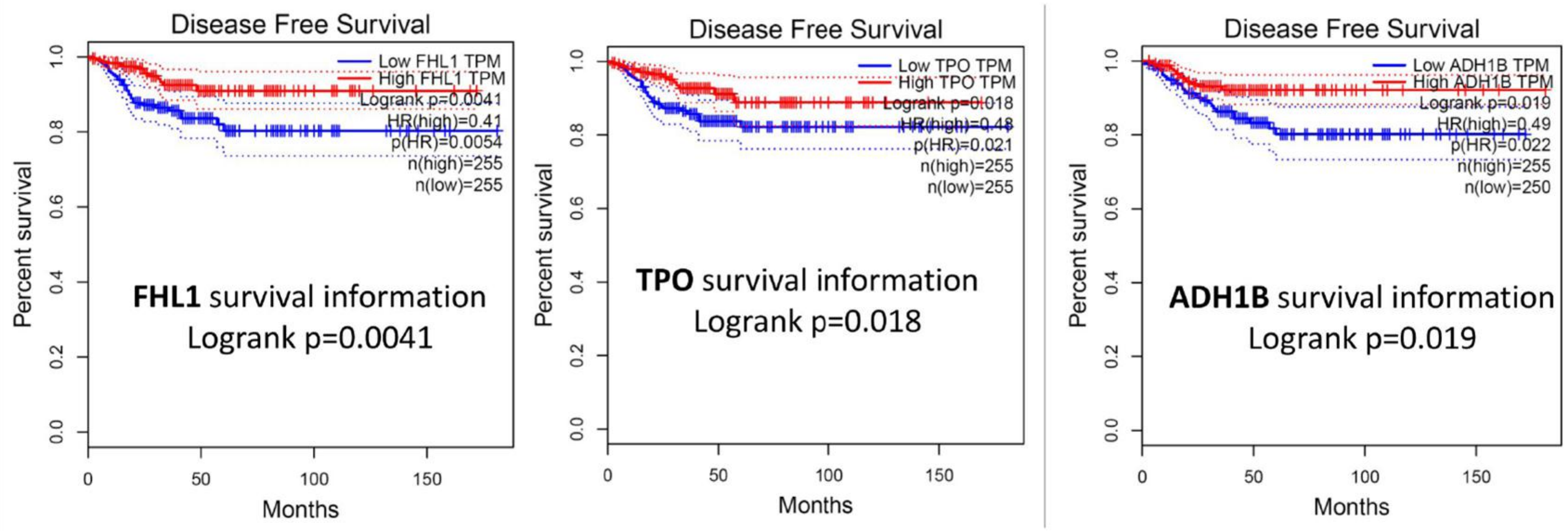
Disease Free Survival
Low FHL1 TPM
High FHL1 TPM
Logrank p=0.0041
HR(high)=0.41
p(HR)=0.0054
n(high)=255
n(low)=255
FHL1 survival information
Logrank p=0.0041
Percent survival
Months
Disease Free Survival
Low TPO TPM
High TPO TPM
Logrank p=0.018
HR(high)=0.48
p(HR)=0.021
n(high)=255
n(low)=255
TPO survival information
Logrank p=0.018
Percent survival
Months
Disease Free Survival
Low ADH1B TPM
High ADH1B TPM
Logrank p=0.019
HR(high)=0.49
p(HR)=0.022
n(high)=255
n(low)=250
ADH1B survival information
Logrank p=0.019
Percent survival
Months

# Fig S4

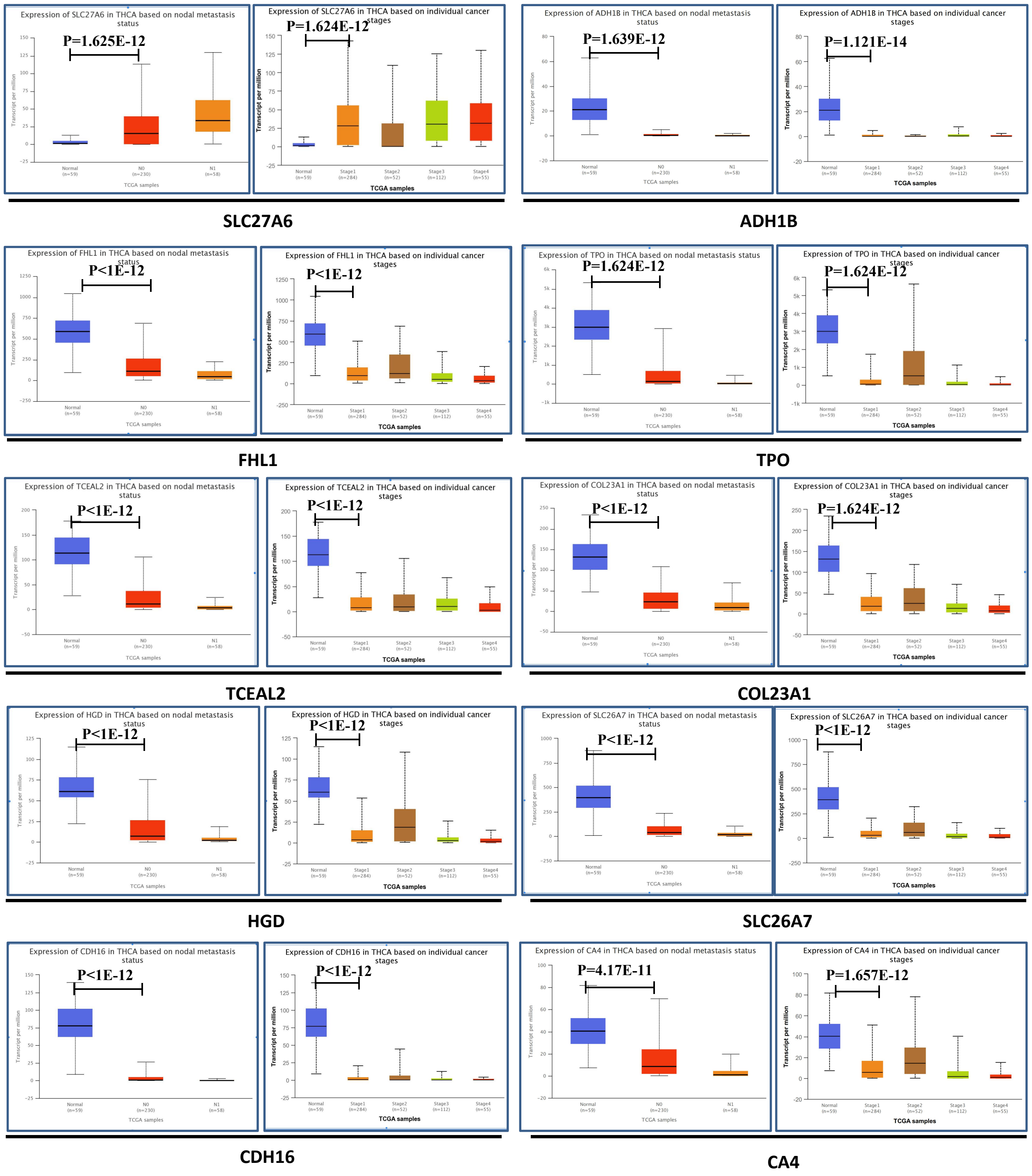

Expression of SLC27A6 in THCA based on nodal metastasis status
P=1.625E-12
Expression of SLC27A6 in THCA based on individual cancer stages
P=1.624E-12
SLC27A6
Expression of ADH1B in THCA based on nodal metastasis status
P=1.639E-12
Expression of ADH1B in THCA based on individual cancer stages
P=1.121E-14
ADH1B
Expression of FHL1 in THCA based on nodal metastasis status
P<1E-12
Expression of FHL1 in THCA based on individual cancer stages
P<1E-12
FHL1
Expression of TPO in THCA based on nodal metastasis status
P=1.624E-12
Expression of TPO in THCA based on individual cancer stages
P=1.624E-12
TPO
Expression of TCEAL2 in THCA based on nodal metastasis status
P<1E-12
Expression of TCEAL2 in THCA based on individual cancer stages
P<1E-12
TCEAL2
Expression of COL23A1 in THCA based on nodal metastasis status
P<1E-12
Expression of COL23A1 in THCA based on individual cancer stages
P=1.624E-12
COL23A1
Expression of HGD in THCA based on nodal metastasis status
P<1E-12
Expression of HGD in THCA based on individual cancer stages
P<1E-12
HGD
Expression of SLC26A7 in THCA based on nodal metastasis status
P<1E-12
Expression of SLC26A7 in THCA based on individual cancer stages
P<1E-12
SLC26A7
Expression of CDH16 in THCA based on nodal metastasis status
P<1E-12
Expression of CDH16 in THCA based on individual cancer stages
P<1E-12
CDH16
Expression of CA4 in THCA based on nodal metastasis status
P=4.17E-11
Expression of CA4 in THCA based on individual cancer stages
P=1.657E-12
CA4
Transcript per million
Normal (n=59)
N0 (n=230)
N1 (n=58)
Stage1 (n=284)
Stage2 (n=52)
Stage3 (n=112)
Stage4 (n=55)
TCGA samples
